# Alterations in the tyrosine and phenylalanine pathways revealed by biochemical profiling in cerebrospinal fluid of Huntington’s disease subjects

**DOI:** 10.1038/s41598-019-40186-5

**Published:** 2019-03-11

**Authors:** Stephanie Herman, Valter Niemelä, Payam Emami Khoonsari, Jimmy Sundblom, Joachim Burman, Anne-Marie Landtblom, Ola Spjuth, Dag Nyholm, Kim Kultima

**Affiliations:** 1 Department of Medical Sciences, Clinical Chemistry, Uppsala University, Uppsala, Sweden; 2 Department of Pharmaceutical Biosciences, Uppsala University, Uppsala, Sweden; 3 Department of Neuroscience, Neurology, Uppsala University, Uppsala, Sweden; 4 Department of Neuroscience, Neurosurgery, Uppsala University, Uppsala, Sweden

## Abstract

Huntington’s disease (HD) is a severe neurological disease leading to psychiatric symptoms, motor impairment and cognitive decline. The disease is caused by a CAG expansion in the huntingtin (HTT) gene, but how this translates into the clinical phenotype of HD remains elusive. Using liquid chromatography mass spectrometry, we analyzed the metabolome of cerebrospinal fluid (CSF) from premanifest and manifest HD subjects as well as control subjects. Inter-group differences revealed that the tyrosine metabolism, including tyrosine, thyroxine, L-DOPA and dopamine, was significantly altered in manifest compared with premanifest HD. These metabolites demonstrated moderate to strong associations to measures of disease severity and symptoms. Thyroxine and dopamine also correlated with the five year risk of onset in premanifest HD subjects. The phenylalanine and the purine metabolisms were also significantly altered, but associated less to disease severity. Decreased levels of lumichrome were commonly found in mutated HTT carriers and the levels correlated with the five year risk of disease onset in premanifest carriers. These biochemical findings demonstrates that the CSF metabolome can be used to characterize molecular pathogenesis occurring in HD, which may be essential for future development of novel HD therapies.

## Introduction

Huntington’s disease (HD) is a severe neurodegenerative disease characterized by psychiatric symptoms, motor impairment and cognitive decline. The disease is caused by a polyglutamine (encoded by the nucleotide triplet CAG) expansion on exon 1 of the huntingtin (HTT) gene, which is dominantly inherited^1^. CAG expansion does not lead to disease until a threshold is reached. At 36 repeats, there is a risk for development of HD, usually presenting late in life. Individuals with 40 repeats or more will invariably develop the disease. A very large number of CAG repeats (60 or more) usually leads to onset before 20 years of age.

Although the molecular mechanisms remain elusive, HD pathogenesis have been proposed to be associated with a combination of toxic gain of function of the mutant HTT (mHTT) protein and loss of function of the wild-type HTT protein, untranslated RNA toxicity^2^, rising instability and oxidative damage of genomic DNA^3^ and CAG repeat associated non-ATG translation^4^, where the impaired systems include mitochondrial function, axonal transport, hormone secretion and neurotrophic support^5,6^. Currently there are no therapies for HD, which typically is fatal within twenty years of onset^7^.

The cerebrospinal fluid (CSF) metabolome is a valuable source to monitor biochemical changes in the central nervous system (CNS) occurring in the events of neurological diseases^8^ and it represents an unexplored compartment in understanding the effects of the HTT mutation in humans. Using metabolomics based methods, disturbed energy metabolism was reported in CSF of premanifest HD transgenic rats compared with wild-type^9^ and in human plasma of HD patients^10^. Also in *post mortem* HD brain tissue (frontal lobe and striatum), changes in energy metabolism as well as phospholipid metabolism was reported^11,12^. CSF is the closest biological compartment to the CNS (except for the brain itself) and should therefore reflect the pathological changes occurring in the brain more accurately than i.e. plasma or serum^13^.

The human CSF metabolome of mHTT carriers has to the best of our knowledge not been investigated before. To fill this gap, we devised a mass spectrometry (MS) based metabolomics approach to investigate changes in the CSF metabolome of mHTT carriers in comparison to controls, as well as differences between the metabolomes of premanifest and manifest HD subjects. To enable monitoring over time, repeated samples were collected for a subset of the premanifest and manifest HD subjects. Finally, the metabolites underlying the differences were connected to biochemical pathways and to measures of disease severity and symptoms.

## Methods

### Ethical approval

The study was approved by the Regional Ethical Review Board in Uppsala, Sweden (Dnr 2012/274 and 2013/278). All participants provided written informed consent before any samples were collected.

### Enrollment of study participants

Thirteen manifest and 13 premanifest mHTT carriers, and 42 controls were enrolled at Uppsala University Hospital. Premanifest HD was defined as individuals with a mHTT CAG expansion (≥36 repeats) and a diagnostic confidence level below four, whereas manifest HD was defined by a similar CAG expansion and diagnostic confidence level of four^14^. Eight of the manifest and seven of the premanifest mHTT carriers donated a second sample at follow-up.

### Clinical assessment

All mHTT carriers were assessed using the total motor score (TMS), total functional capacity (TFC)^15^ as well as the cognitive performance measures: Stroop interference (SI), Stroop color (SC), Stroop word reading (SWR)^16^, category verbal fluency (CVF), verbal fluency letters (VFL) and the symbol digit modalities test (SDMT). The number of CAG repeats and age were used to calculate the disease burden score (DBS) for all mHTT carriers and the five year risk of onset for the premanifest HD subjects^17^.

### CSF handling and storage

Samples were collected and stored in accordance with the guidelines for CSF collection, formed by the BioMS-eu network^18^. The control samples were collected by lumbar punctures made in routine health care. Patients were asked to donate 3 mL for research purposes. If consent was given the samples were brought to the laboratory within 30 minutes and then entered a semi-automatic process of spinning, aliquoting and freezing. The samples were centrifuged at 250 g for five minutes, stored in polypropylene tubes in aliquots of 240 µL at −80 °C until analyzed.

### Metabolite extraction

CSF samples were thawed on ice and 100 µL was mixed with 410 µL ice-cold methanol (MeOH) supplemented with internal standards at a final concentration of 0.25 µM. The samples were vortexed for 15 s and incubated at −20 °C for 30 minutes, followed by centrifugation at 20400 g for 12 minutes at 4 °C. The supernatants were transferred to fresh Eppendorf tubes which were dried using a centrifugal vacuum concentrator (overnight).

Upon analysis the dried samples were reconstituted in 100 µL 5% MeOH, 0.1% formic acid and 94.9% deionized MilliQ water. 10 µL of each sample was pooled to create a quality control (QC) sample to be injected repeatedly to monitor the MS performance by ensuring that the internal standards were present and in correct magnitude.

### Mass spectrometry analysis

The samples were injected twice for duplicates, in a constrained randomized order. A QC injection was done every 16^th^ sample and blank injection every 8^th^. Finally, a 2-fold serial dilution series ranging from 0.5 to 32.0 µL QC was injected. In addition, MS/MS analysis was performed on pooled samples containing all samples within the three study groups (premanifest HD, manifest HD and controls) and on the global pool (the QC sample). The analysis was performed on a Thermo Ultimate 3000 HPLC and Thermo Q-Exactive Orbitrap mass spectrometer. Detailed description of the analysis can be found in the Supplementary information.

### Quantification and quality assessment

The acquired raw data was converted to an open source format (mzML). Peaks were centroided by *msconvert* from ProteoWizard^19^ and preprocessed using the following pipeline within the KNIME platform^20^: the peak-picked data was quantified by *FeatureFinderMetabo*^21^ and features were linked across samples using *featureLinkerUnlabelledQT*^22^, allowing 10 s retention time tolerance and 5 ppm mass deviation (performed irrespective of charge state across the samples). The non-default parameters used can be found as Supplementary Table S1.

The quantified data was loaded into the statistical software environment R v3.4.0^23^. Blank filtering was performed according to our previously introduced pipeline^8,24^. A quality filtering procedure was performed using the dilution series keeping only spectral features with at least an absolute Pearson correlation of 0.3 with the injection volume. To stabilize the variance, the intensity values were replaced by its log_2_ value and potential sample outliers were detected and removed by calculating the total ion count (TIC) of each sample. Samples with a TIC less than 60% of the average TIC were seen as outliers and removed from the study. One sample replicate was removed. To further reduce the intensity decay over run time, the spectral features were normalized using normalize cyclic loess from the R-package *limma*^25^. The in-between-replicate correlation was calculated (minimum replicate correlation achieved was 0.92) and the replicates averaged. Finally, the coverage within metabolites were required to be at least 75% for inclusion and the remaining missing values were replaced by its average feature value.

### Metabolite identification

All metabolic features with a 75% coverage were matched against an *in house* library of characterized metabolites using a 15 ppm mass tolerance and a 20 s time window. Metabolites (identified metabolic features) of interest were manually curated on MS/MS level when available. Identities confirmed by m/z and elution time of the pure standards and by MS/MS fragmentation pattern were depicted as verified on validation level 2. Identities confirmed only by m/z and elution time of the pure standards were depicted as verified on validation level 1. Identities of metabolites with available MS/MS fragmentation that did not match the authentic standard were rejected.

### Statistical analysis

*Post hoc* comparisons were performed for age, number of CAG repeats, gender, DBS, the measures of disease severity and time intervals between repeated sampling for premanifest and manifest HD subjects in the first and second sampling sets, respectively. For variables not significantly different from normal distribution, Welch’s *t*-test was used, otherwise the non-parametric Mann-Whitney test was used. The Shapiro-Wilks Normality test was used to assess normality of variables. For the categorical variable gender, a Chi-squared test was used. To compare symptom severity before and after follow-up, paired *t*-tests were performed comparing the HD symptom metrics including DBS at the first and second sampling occasions. A p-value lower than 0.05 was considered statistically significant.

In order to eliminate age as a confounder, age was corrected for using linear detrending based on only controls. The controls had been selected to completely span the age distribution of all mHTT carriers. Metabolic features with a significant age dependence (p-value < 0.05) were corrected by fitting a linear regression model (R function *lm*) for each metabolic feature in the controls, with age as the explanatory variable. The age coefficient was extracted from the model and used to correct the metabolic levels in all individuals^26^.

Principal component analysis (PCA) was performed using the function *pca* from the R package *mixOmics*^27^ using all metabolic features. To specifically target inter-group differences, supervised multivariate analysis using partial least square discriminant analysis (PLS-DA) was performed on identified metabolites. The data was scaled (zero mean, unit variance) and three PLS-DA models were trained on the first sampling set, comparing two groups at a time using the R package *ropls*^28^. The most significant variables were obtained using the “Variable Importance in the Projection” (VIP). To assess model performance the quality metrics R^2^ and Q^2^ were extracted and the area under the receiver operating characteristic (AUROC) curve was computed using the R-package *pROC*^29^. To ensure reproducibility, VIP scores and AUROC values including the receiver operating characteristic (ROC) curves were collected through a 5-fold cross-validation (CV) repeated ten times, where the number of components were fixed to one. Briefly, the 5-fold CV divides the data into five balanced groups using stratified sampling. Four of these groups are used for training the model, while the fifth is used for validation and performance estimation. The procedure is repeated five times, so that each group may act as validation set. Variables with an average VIP score above 1.0 were seen as significantly altered and subjected to a Welch’s *t*-test, accounting for unequal variance and unequal sample size. The p-values were adjusted for multiple comparisons using false discovery rate (FDR). A FDR value < 0.1 was considered significant. Finally, paired *t*-tests were done comparing the longitudinal first and second samplings of premanifest and manifest HD subjects respectively. A p-value < 0.05 was considered statistically significant.

Altered metabolites (VIP > 1.0) with an available HMDB code from each model were separately subjected to a pathway analysis using MetaboAnalyst^30^ and the *Homo sapiens* pathway library. The significance levels were based on the hypergeometric test and the relative-betweenness centrality was used to compute the pathway impact. A FDR value < 0.1 was considered statistically significant.

Finally, Spearman’s rank correlation analyses were performed between all altered metabolites and the measures of disease severity, including DBS and the five year risk of onset. A p-value < 0.05 was considered statistically significant. A clustering analysis was performed on the correlation values of the metabolites using the R function *hclust* (default parameters). The Euclidean distance was used as measure of similarity and computed using the R function *dist*.

## Results

### Research subjects characteristics

**Figure 1:**
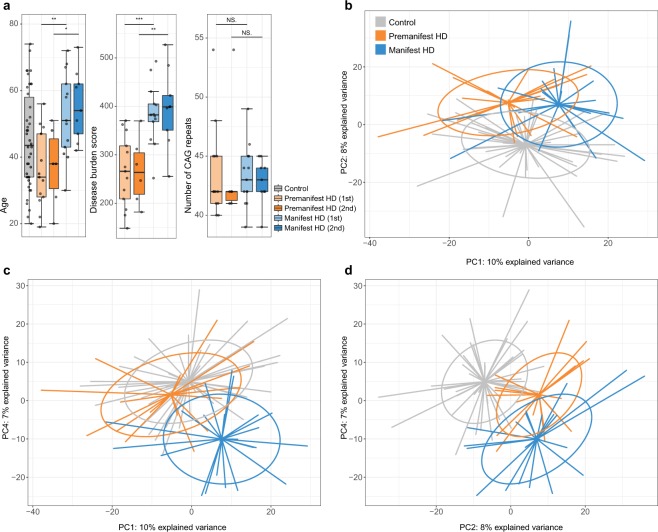
(**a**) The age, disease burden score and CAG expansion distributions in the study cohort stratified on diagnostic groups and sampling occasion. *p < 0.05, **p < 0.01 and ***p < 0.001. (**b–d**) Age-adjusted principal component analysis (PCA) on all metabolic features. Separation between all study groups can be seen in (**b**) PC1 vs. PC2 and (**d**) PC2 vs. PC4 whereas a clear separation of the manifest HD patients can be seen in (**c**) PC1 vs. PC4.

As expected, the manifest HD patients were significantly older compared with the premanifest mHTT carriers, had higher DBS and TMS, lower TFC as well as lower cognitive performance, whereas no significant difference in gender distribution or the number of CAG repeats were present (Table 1, Fig. 1a). No significant differences were neither found in gender distribution of the premanifest and manifest HD subjects compared with the control subjects.

**Table 1:** Clinical and demographic data including follow-up data of the mHTT carriers. Only antidepressants and antipsychotics have been included in the number of subjects on medication.

*Cohort*	Controls	Premanifest HD		Manifest HD	
*n*	42	13		13	
Female/Male	27/15	6/7		5/8	
On medication, *n*	6	1		8	
Age*, median(range)	44(20–74)	34(19–56)		51(30–72)	
CAG, median(range)	n/a	42(40–54)		43(39–49)	
5 year risk of onset, % median(range)	n/a	23(1–46)		n/a	
DBS*, mean(±SD)	n/a	265(±73.7)		385(±62.9)	
TFC*, mean(±SD)	n/a	13(±0.0)		9.5(±3.50)	
TMS*, median(range)	n/a	1(0–4)		29(8–64)	
CVF*, mean(±SD)	n/a	22(±3.7)		12(±5.3)	
VFL*, mean(±SD)	n/a	42(±15.3)		17(±10.1)	
SDMT*, mean(±SD)	n/a	48(±12.5)		20(±9.94)	
SI*, median(range)	n/a	47(38–62)		22(6–36)	
SWR* mean(±SD)	n/a	88(±15.7)		48(±22.2)	
SC*, mean(±SD)	n/a	69(±11.2)		33(±14.9)	
***Follow-up***		**Premanifest HD**		**Manifest HD**	
*n*		7		8	
Female/Male		3/4		3/5	
On medication, *n*		3		5	
Transitioned, *n*		1		n/a	
Sampling interval in months, median(range)		12(11.2–44.6)		28(10.4–44.2)	
***Sampling*** ^**a**^		**1** ^**st**^	**2** ^**nd**^	**1** ^**st**^	**2** ^**nd**^
5 year risk of onset^$^, % median(range)		14(1–30)	17.5(2–33)	n/a	n/a
DBS^$^, mean(±SD)		254(±69.2)	267(±69.0)	380(±79.6)	396(±86.0)
TFC, mean(±SD)		13(±0.0)	13(±0.0)	9.8(±3.20)	8.0(±3.93)
TMS, median(range)		1.5(0–4)	1.5(0–5)	28(8–64)	32.5(17–60)
CVF, mean(±SD)		22(±3.11)	19(±7.28)	13(±3.31)	12(±7.95)
VFL, mean(±SD)		38(±12.8)	40(±11.1)	16.7(±11.3)	16.9(±12.5)
SDMT, mean(±SD)		46(±17.0)	46(±14.3)	24(±3.99)	24(±11.6)
SI, median(range)		45.5(38–50)	46(34–57)	22(6–31)	19(11–28)
SWR, mean(±SD)		85(±19.6)	87(±28.5)	50(±17.4)	51(±11.9)
SC, mean(±SD)		72(±14.2)	66(±13.6)	36(±10.4)	33(±9.25)

*A significant difference between premanifest and manifest HD subjects. ^$^A significant difference between the 1^st^ and 2^nd^ samplings for both premanifest and manifest HD subjects. ^a^The phenoconverter was excluded in comparisons between 1^st^ and 2^nd^ samplings. DBS: disease burden score; TFC: total functional capacity; 5yrisk: the five year risk of onset; TMS: total motor score; SC: Stroop color; SWR: Stroop word reading; SI: Stroop interference; CVF: category verbal fluency; VFL: verbal fluency letters; SDMT: symbol digit modalities test.

A second CSF sample was collected from seven premanifest and eight manifest HD subjects on average 20 months (range: 10.4–44.6) after the first sample was taken, showing that the premanifest and manifest HD subjects had higher DBS at follow-up. The premanifest subjects also displayed an increased five years risk of onset. No other significant changes were found.

One of the premanifest subjects and six of the manifest patients were on antidepressants. Two of the manifest patients were on antipsychotics. Six controls were on antidepressants (Table 1). At follow-up, one of the premanifest HD subjects had developed manifest HD and started taking antidepressants. In addition, one premanifest and one manifest HD patient had started taking antidepressants.

### The CSF metabolome distinguished mHTT carriers from controls

In total, 3701 metabolic features were matched across all samples with a 75% coverage. Out of these, 94 metabolites were successfully identified using an *in house* library. In total, 22% of all metabolic features and 28(30%) of the identified metabolites were age dependent and therefore corrected for age in all subjects.

A PCA based on all metabolic features revealed that the premanifest and manifest HD groups were separated from each other over PC1 and from the controls over PC2 that explained ten and eight percentage of the variance each (Fig. 1b). When including PC4, explaining seven percent variance, the manifest HD patients were clearly separated from the rest, indicating a specific biochemical signature of this group (Fig. 1c,d). We also performed a PCA limited to the 94 identified metabolites, which separated the groups to a lesser degree (see Supplementary Fig. S1).

**Figure 2:**
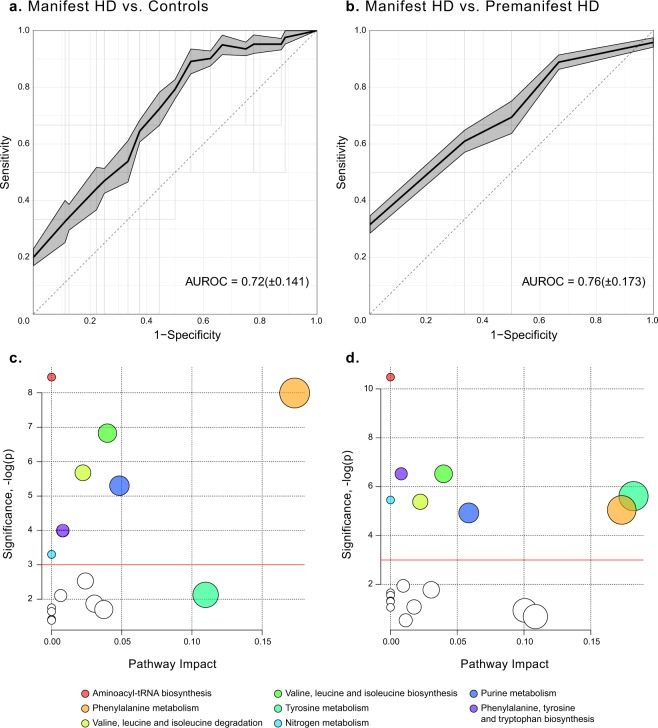
Average ROC curves with corresponding average and standard deviation of the AUROC for the PLS-DA models comparing (**a**) manifest HD patients with controls and (**b**) manifest HD patients with premanifest HD subjects. The shadowed areas indicate the standard error of the mean of the sensitivity and 1-specificity. (**c**,**d**) Pathway analyses of the altered metabolites between manifest HD compared with (**c**) controls and (**d**) premanifest HD. The size of the node indicates the pathway impact (similar to the x-axis) computed by the relative-betweenness centrality and the color corresponds to the corresponding pathway. Pathways that were non-significant in both comparisons have been colored white. The red lines indicate the significance level of a p-value = 0.05.

In order to extract altered metabolites, PLS-DA models were used to compare premanifest HD, manifest HD and controls. The first model (manifest HD vs. controls) achieved quality metrics of R^2^ = 0.76: p < 0.05, Q^2^ = 0.30: p < 0.05 and an average AUROC of 0.72(± 0.141), Fig. 2a. The second model (manifest vs. premanifest HD) achieved quality metrics of R^2^ = 0.54: p < 0.85, Q^2^ = 0.16: p < 0.15 and an average AUROC of 0.76(± 0.173), Fig. 2b. Both indicating segregations between the groups. The third model, comparing the premanifest HD subjects with controls were found to be non-predictive (Q^2^ < 0).

### Altered CSF metabolites in manifest HD patients compared with controls

Comparing manifest HD patients with controls, 24 metabolites were significantly altered, Table 2. The univariate tests demonstrated that ten of these were independently altered, of which lumichrome, xanthine, N-acetylproline, isoleucine and L-DOPA remained significant after correcting for multiple comparisons. Xanthine, O-succinyl-homoserine, N-acylproline, glutarylcarnitine, tyrosine, 5-methylcytosine had been corrected for a significant age dependence. In the eight manifest patients, from which a second longitudinal sample was collected, no differences were found between first and follow-up for any of the altered metabolites.

**Table 2:** Altered metabolites with an average VIP score greater than 1.0 from the PLS-DA comparing manifest HD patients (HD) with controls (C).

Metabolite	HMDB	VIP mean(95% CI)	log_2_ FC HD-C	p-value	FDR	Validation level
Lumichrome	—	2.56(2.47, 2.64)	−1.02	**6.3 × 10** ^**−3**^	**0.047**	2
Xanthine*	HMDB00292	2.33(2.28, 2.38)	0.28	**3.9 × 10** ^**−3**^	**0.047**	2
O-succinyl-homoserine*	YMDB00917	1.94(1.83, 2.05)	−0.38	**0.043**	0.101	1
N-acetylproline*	—	1.90(1.81, 1.99)	0.25	**6.0 × 10** ^**−3**^	**0.047**	1
Phenylacetate	HMDB00209	1.81(1.72, 1.90)	0.20	**0.030**	0.100	2
Isoleucine	HMDB00172	1.81(1.72, 1.89)	−0.51	**7.8 × 10** ^**−3**^	**0.047**	2
L-DOPA	HMDB00181	1.77(1.69, 1.86)	−0.34	**0.015**	**0.070**	1
Leucine	HMDB00687	1.76(1.66, 1.86)	−0.23	**0.033**	0.100	2
Corticosterone	HMDB01547	1.52(1.40, 1.64)	−0.70	0.066	0.101	1
Ophthalmate	HMDB05765	1.44(1.35, 1.53)	−0.34	0.065	0.101	1
Salicylate	HMDB01895	1.42(1.33, 1.51)	−0.46	**0.031**	0.100	1
Phenylalanine	HMDB00159	1.42(1.31, 1.52)	−0.12	0.087	0.116	2
Deoxyuridine	HMDB00012	1.36(1.27, 1.46)	−0.20	0.075	0.106	1
Valine	HMDB00883	1.33(1.24, 1.43)	−0.16	0.059	0.101	2
Creatinine	HMDB00562	1.33(1.23, 1.42)	0.16	0.053	0.101	2
Inosine	HMDB00195	1.32(1.22, 1.43)	0.14	0.147	0.160	2
Cyclic AMP	HMDB00058	1.31(1.19, 1.42)	0.21	0.106	0.127	1
Hypoxanthine	HMDB00157	1.29(1.23, 1.36)	0.20	0.059	0.101	2
Glutarylcarnitine*	HMDB13130	1.24(1.16, 1.33)	−0.24	0.114	0.130	2
Phosphocreatine	HMDB01511	1.16(1.08, 1.25)	0.27	0.067	0.101	1
Aldosterone/Cortisone	—/HMDB02802	1.13(1.07, 1.19)	−0.36	**0.044**	0.101	1
Tyrosine*	HMDB00158	1.13(1.04, 1.21)	−0.11	0.095	0.120	2
5-methylcytosine*	HMDB02894	1.11(1.01, 1.22)	0.11	0.163	0.170	1
1-methyladenosine	HMDB03331	1.02(0.91, 1.13)	−0.45	0.198	0.198	1

A positive log_2_ fold change (FC) HD-C indicates an increase in manifest HD patients compared with controls and vice versa. p-values < 0.05 and FDR values < 0.1 have been bolded. *Metabolites with a demonstrated age dependence that have been corrected. Identities confirmed by m/z and elution time of the internal standards and by MS/MS fragmentation pattern (validation level 2). Identities confirmed by m/z and elution time of the internal standards (validation level 1).

The pathway analysis revealed five biochemical pathways that were affected in manifest HD compared with controls: aminoacyl-tRNA biosynthesis; the phenylalanine metabolism; valine, leucine and isoleucine biosynthesis; valine, leucine, isoleucine degradation and the purine metabolism, where the phenylalanine metabolism showed the highest impact (Fig. 2c, Table 3). A complete table of the pathway analysis can be found as Supplementary Table S2.

**Table 3:** Results from the pathway analysis based on the altered metabolites in manifest HD patients compared with controls. p-values < 0.05 and FDR values < 0.1 have been bolded.

Pathway	Coverage	p-value	FDR	Impact
Aminoacyl-tRNA biosynthesis	5/75	**2.1 × 10** ^**−4**^	**0.014**	0.0
Phenylalanine metabolism	4/45	**3.4 × 10** ^**−4**^	**0.014**	0.173
Valine, leucine and isoleucine biosynthesis	3/27	**1.1 × 10** ^**−3**^	**0.029**	0.040
Valine, leucine and isoleucine degradation	3/40	**3.4 × 10** ^**−3**^	**0.069**	0.022
Purine metabolism	4/92	**5.0 × 10** ^**−3**^	**0.080**	0.048
Phenylalanine, tyrosine and tryptophan metabolism	2/27	**0.018**	0.246	0.008
Nitrogen metabolism	2/39	**0.037**	0.420	0.0

### Altered CSF metabolites in manifest compared with premanifest HD subjects

Comparing manifest HD patients with premanifest subjects revealed 28 metabolites as significantly altered, Table 4. Out of these, 14 were also of importance in distinguishing between manifest HD patients and controls. The univariate tests demonstrated that 11 metabolites were perturbed, of which L-DOPA, xanthine, ophthalmate, creatinine, tyrosine, 5-hyproxytryptophan, adenosine and phenylalanine remained significant after correcting for multiple comparisons. In addition to those previously mentioned, 4-acetamidobutanoate and S-adenosylhomocysteine had been corrected for an age dependence.

**Table 4:** Altered metabolites with an average VIP score greater than 1.0 from the PLS-DA comparing premanifest (pHD) and manifest (HD) subjects.

Metabolite	HMDB	VIP mean(95% CI)	log_2_ FC HD-pHD	p-value	FDR	log_2_ FC HD_2_-HD_1_	log_2_ FC pHD_2_-pHD_1_	Validation level
L-DOPA	HMDB00181	2.14 (2.08, 2.20)	−0.53	**2.5 × 10** ^**−3**^	**0.041**	n.s.	n.s.	1
Xanthine*	HMDB00292	2.10 (2.05, 2.14)	0.29	**3.8 × 10** ^**−3**^	**0.041**	n.s.	n.s.	2
Ophthalmate	HMDB05765	2.05 (1.98, 2.13)	−0.81	**4.4 × 10** ^**−3**^	**0.041**	n.s.	−0.44	1
Creatinine	HMDB00562	1.81 (1.75, 1.87)	0.23	**0.013**	**0.082**	n.s.	n.s.	2
Tyrosine*	HMDB00158	1.78 (1.70, 1.86)	−0.22	**0.015**	**0.082**	n.s.	n.s.	2
5-hydroxytryptophan	HMDB00472	1.74 (1.65, 1.84)	−0.61	**0.018**	**0.084**	n.s.	n.s.	1
Adenosine	HMDB00050	1.71 (1.63, 1.78)	0.49	**0.021**	**0.084**	n.s.	n.s.	1
Phenylalanine	HMDB00159	1.64 (1.59, 1.69)	−0.18	**0.027**	**0.096**	n.s.	**0.04**	2
Phenylacetate	HMDB00209	1.55 (1.46, 1.65)	0.25	**0.034**	0.107	n.s.	n.s.	2
DHEAS	—	1.53 (1.47, 1.59)	−0.99	**0.040**	0.112	n.s.	n.s.	2
Thyroxine	HMDB00248	1.49 (1.40, 1.58)	−0.37	**0.045**	0.114	n.s.	n.s.	1
Glutarylcarnitine*	HMDB13130	1.38 (1.30, 1.46)	−0.33	0.063	0.143	n.s.	n.s.	2
O-succinyl-homoserine*	YMDB00917	1.36 (1.27, 1.46)	−0.39	0.071	0.143	n.s.	n.s.	1
Adenine	HMDB00034	1.35 (1.24, 1.45)	0.25	0.080	0.143	n.s.	n.s.	1
Isoleucine	HMDB00172	1.34 (1.25, 1.43)	−0.32	0.079	0.143	n.s.	n.s.	2
Aldosterone/Cortisone	—/HMDB02802	1.31 (1.21, 1.42)	−0.27	0.082	0.143	n.s.	n.s.	1
4-quinolinecarboxylate	—	1.26 (1.17, 1.36)	−0.22	0.097	0.159	n.s.	**−0.25**	1
O-acetylcarnitine	HMDB00201	1.20 (1.12, 1.28)	−0.25	0.108	0.164	**−0.22**	n.s.	1
4-acetamidobutanoate*	HMDB03681	1.18 (1.06, 1.30)	0.19	0.117	0.164	n.s.	n.s.	2
Hypoxanthine	HMDB00157	1.17 (1.09, 1.26)	0.18	0.117	0.164	n.s.	n.s.	2
N,N,N-trimethyllysine	HMDB01325	1.17 (1.07, 1.28)	0.21	0.123	0.164	n.s.	**0.18**	1
Leucine	HMDB00687	1.13 (1.04, 1.22)	−0.16	0.136	0.166	n.s.	n.s.	2
Dopamine	HMDB00073	1.13 (1.01, 1.25)	−0.28	0.137	0.166	n.s.	n.s.	1
N-acetylalanine	HMDB00766	1.08 (0.98, 1.17)	0.14	0.143	0.166	n.s.	n.s.	1
Tryptophan [M-H]	HMDB00929	1.07 (0.99, 1.16)	−0.19	0.154	0.172	n.s.	n.s.	2
Valine	HMDB00883	1.03 (0.94, 1.13)	−0.11	0.181	0.180	n.s.	n.s.	2
Tryptophan [M + H]	HMDB00929	1.03 (0.94, 1.13)	−0.15	0.170	0.180	n.s.	n.s.	2
S-adenosylhomocysteine*	HMDB00939	1.03 (0.92, 1.14)	0.20	0.175	0.180	n.s.	n.s.	1

A positive log_2_ fold change (FC) HD-pHD indicates an increase in manifest HD patients compared with premanifest subjects and vice versa. A positive log_2_ FC HD_2_-HD_1_ indicates an increase over time in manifest HD patients and similar for log_2_ FC pHD_2_-pHD_1_ in premanifest HD subjects. Non-significant FC (p < 0.05) have been marker with n.s. p-values < 0.05 and FDR values < 0.1 have been bolded. *Metabolites with a demonstrated age dependence that have been corrected. Identities confirmed by m/z and elution time of the internal standards and by MS/MS fragmentation pattern (validation level 2). Identities confirmed by m/z and elution time of the internal standards (validation level 1).

There was a significant longitudinal decrease in the levels of O-acetylcarnitine in the manifest HD patients. Significantly altered levels of ophthalmate, phenylalanine, 4-quinolinecarboxylate and N,N,N-trimethyllysine were found in the six premanifest HD subjects (excluding the phenoconverting patient) where a longitudinal sample had been collected.

Using pathway analysis, eight biochemical pathways were significantly perturbed between manifest and premanifest HD subjects: aminoacyl-tRNA biosynthesis; the phenylalanine, tyrosine and tryptophan biosynthesis; valine, leucine and isoleucine biosynthesis; the tyrosine metabolism; the nitrogen metabolism; valine, leucine and isoleucine degradation; the phenylalanine metabolism and the purine metabolism (Fig. 2d, Table 5), where tyrosine metabolism and phenylalanine metabolism showed the highest impacts. A complete table of the pathway analysis can be found as Supplementary Table S3.

**Table 5:** Results from the pathway analysis based on the altered metabolites in manifest HD patients compared with premanifest HD subjects. p-values < 0.05 and FDR values < 0.1 have been bolded.

Pathway	Coverage	p-value	FDR	Impact
Aminoacyl-tRNA biosynthesis	6/75	**2.8 × 10** ^**−5**^	**2.2 × 10** ^**−3**^	0.0
Phenylalanine, tyrosine and tryptophan biosynthesis	3/27	**1.5 × 10** ^**−3**^	**0.039**	0.008
Valine, leucine and isoleucine biosynthesis	3/27	**1.5 × 10** ^**−3**^	**0.039**	0.040
Tyrosine metabolism	4/76	**3.7 × 10** ^**−3**^	**0.061**	0.182
Nitrogen metabolism	3/39	**4.3 × 10** ^**−3**^	**0.061**	0.0
Valine, leucine and isoleucine degradation	3/40	**4.6 × 10** ^**−3**^	**0.061**	0.022
Phenylalanine metabolism	3/45	**6.4 × 10** ^**−3**^	**0.073**	0.173
Purine metabolism	4/92	**7.3 × 10** ^**−3**^	**0.073**	0.059

### Metabolite-to-pathway linkages for all altered metabolites

**Figure 3:**
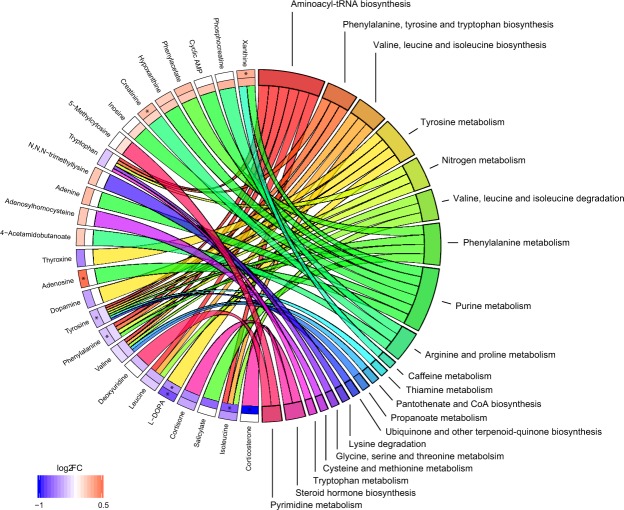
Linkages between altered metabolites and biochemical pathways. The altered metabolites have been linked with pathways as color-coded ribbons. Blue-to-red coding next to the metabolites depicts the magnitude of the log_2_ fold change (FC), where the inner layer represents FC in manifest compared with premanifest HD (HD-pHD) and the outer in comparison with controls (HD-C). Significant FC with an FDR < 0.1 have been marked with ‘*’.

Disregarding pathway impacts, investigating only linkages between all altered metabolites and biochemical pathways revealed that six metabolites have been connected to purine metabolism and aminoacyl-tRNA biosynthesis respectively, and four metabolites to tyrosine and phenylalanine metabolisms respectively (Fig. 3). Additionally, tryptophan, tyrosine, phenylalanine and valine were recurrent in multiple pathways.

### Metabolite association to disease severity

**Figure 4:**
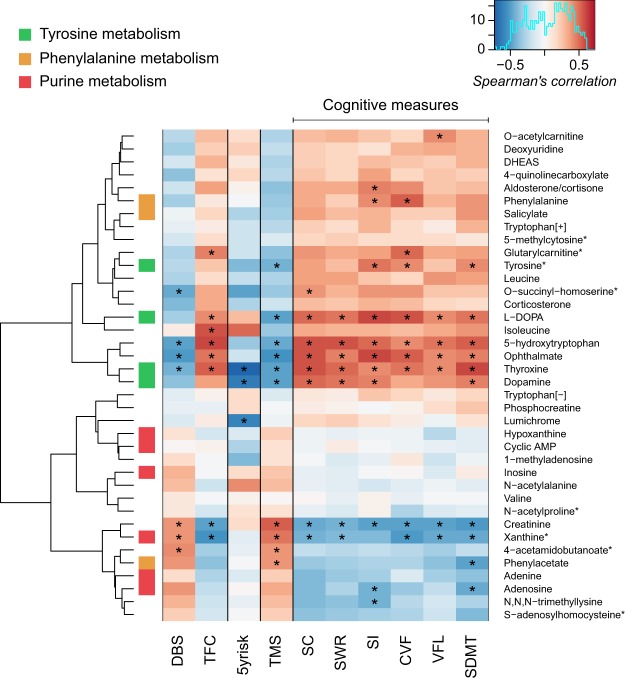
Associations between the biochemical changes and HD symptoms, where a ‘*’ indicates a corresponding p-value < 0.05. Hierarchical clustering has been performed on the correlation values where metabolites from the tyrosine metabolism, phenylalanine metabolism as well as the purine metabolism have been marked. DBS: disease burden score; TFC: total functional capacity; 5yrisk: the five year risk of onset; TMS: total motor score; SC: Stroop color; SWR: Stroop word reading; SI: Stroop interference; CVF: category verbal fluency; VFL: verbal fluency letters; SDMT: symbol digit modalities test. *Metabolites with a demonstrated age dependence that have been corrected.

To investigate the effects of disease progression, the associations between CSF levels of altered metabolites and measures of disease severity were investigated in all mHTT carriers and to the five year risk of onset in premanifest HD subjects. The hierarchical clustering revealed a cluster consisting of thyroxine and dopamine from the tyrosine metabolism as well as 5-hydroxytryptophan and ophtalmate that displayed positive correlations to the cognitive measurements and TFC, and negative correlations to DBS, TMS and to the five year risk of onset. Another cluster consisting of creatinine, xanthine, adenine and adenosine from the purine metabolism as well as phenylacetate from the phenylalanine metabolism correlated positively with DBS and TMS and negatively with the cognitive measurements as well as TFC (Fig. 4). Notably, all metabolites from the tyrosine metabolism depicted associations to disease severity and HD symptoms, where thyroxine in particular showed significant correlations to all measures of disease severity, DBS and the five year risk of onset. The correlation values can be found in Supplementary Table S4.

The CSF levels of creatinine, ophthalmate and 5-hydroxytryptophan correlated with all measures of disease severity and DBS, but not to the five year risk of onset. L-DOPA correlated with all cognitive measurements as well as TFC and TMS, whereas xanthine correlated with all measures of disease severity except for SI and the five year risk of onset. Dopamine displayed significant correlations to the five year risk of onset as well as to TMS, SC, SWR, SI and SDMT. Finally, tyrosine displayed significant correlations to TMS, SI, CVF and SDMT, while lumichrome only displayed a significant correlation to the five year risk of onset in premanifest HD subjects.

## Discussion

There is an unmet clinical need for understanding the pathological events occurring in HD and objective markers to monitor disease progression and treatment response are needed. Using CSF as an explorative source, we found multiple biological pathways including tyrosine metabolism, phenylalanine metabolism and purine metabolism that were deranged in manifest HD patients compared with subjects with premanifest HD and controls. In addition, the measures of disease severity enabled us to connect these changes to HD symptoms and the five year risk of onset in premanifest HD.

The tyrosine metabolism, including tyrosine, thyroxine, L-DOPA and dopamine, showed the highest impact based on the pathway analysis and may thus play a central role in disease progression. Thyroxine demonstrated reduced levels in manifest compared with premanifest HD subjects, moderate to strong associations to all measures of disease severity and lower thyroxine levels were associated to an increased five year risk of onset in premanifest HD subject. Importantly, thyroxine was not associated to age in the control group (r = 0.03, p-value = 0.84), thus eliminating age as a potential confounder^31^. In serum, the results for thyroxine levels in mHTT carriers have been inconclusive, reporting no significant change between premanifest and manifest HD^32^ as well as increased levels in HD compared with controls^33^. Reduced thyroxine levels in CSF have been reported in other neurodegenerative disorders such as Alzheimer’s disease (AD)^34^ and tyrosine has been reported decreased in *post mortem* HD brain tissue (frontal lobe and striatum)^12^. In addition, tyrosine metabolism has as a common denominator been found to be perturbed in both AD and Parkinson’s disease (PD)^35^. Thyroid hormones, including thyroxine, are synthesized and released by the thyroid gland and then transported into the CNS^36^. They are mainly involved in the regulation of energy homeostasis and are important for normal brain function. Previously it has been shown that the wild-type HTT protein binds to thyroid hormone receptor-ɑ1 (TRɑ1)^37^, a nuclear receptor for the deiodinated product of thyroxine (T_4_), triiodothyronine (T_3_), which may be a reason for the altered tyrosine metabolism.

Dopamine and L-DOPA are part of the dopaminergic pathway in tyrosine metabolism where dopamine is produced by its precursor L-DOPA, which further is synthesized from tyrosine and phenylalanine, all of which were shown to be reduced in manifest HD patients and were to various degrees associated with disease severity. Similarly to thyroxine, dopamine levels also correlated with the five year risk of onset. These observations suggests a derangement in tyrosine metabolism and more specifically in the dopaminergic pathway in HD, even though the role is still not well understood and previous findings have been inconclusive, as reviewed by Byrne and Wild^38^.

The phenylalanine metabolism also achieved high impact and significance in the pathway analyses, where phenylalanine, salicylate and tyrosine were reduced and phenylacetate increased in manifest compared with premanifest HD and/or controls. The phenylalanine metabolism has a significant role in neurotransmitter regulation and has previously been reported to be affected in both AD and amyotrophic lateral sclerosis (ALS)^35^.

The amino acids phenylalanine, valine, isoleucine and leucine are part of the aminoacyl-tRNA biosynthesis and were all reduced in manifest HD compared with premanifest and controls, but showed only moderate correlations to measures of disease severity. Decreased levels of these large neutral amino acids have previously been reported in *post mortem* brain tissue, CSF and/or serum/plasma in HD subjects on multiple occasions and suggested to be a result of a deficiency in the energy metabolism^10,12,39–42^. Similarly, the valine, leucine and isoleucine degradation pathway, herein perturbed in manifest compared with premanifest HD and controls, has previously been found to be affected in PD^35^.

Moreover, the purine metabolism was found to be significantly altered in manifest compared with premanifest HD and controls. Previously, this has also been found in AD, ALS and PD and therefore suggested to be an alternative pathway for neurodegenerative diseases to overcome the inadequate glucose supply and the deficient energy metabolism^35^. Xanthine and its precursor hypoxanthine was found to be increased in manifest compared with premanifest HD and controls, adenosine and adenine was increased in manifest compared with premanifest HD, and cyclic AMP and inosine was raised in manifest HD compared with controls, which are all part of the purine metabolism. Increased adenosine levels have previously been demonstrated in the striatum of transgenic HD mice and suggested to be a consequence of the mitochondrial dysfunction^43^, whereas the remaining compounds are novel findings in HD.

Intriguingly, lumichrome displayed a twofold decrease in manifest HD patients compared with controls, but no difference between manifest and premanifest HD (see Supplementary Fig. S2). Comparing mHTT carriers (manifest and premanifest HD subjects) to controls supports the finding that decreased levels of lumichrome are typical to mHTT carriers (mHTT carriers-C log_2_ FC = −0.87, p-value < 0.0003, AUROC = 0.77). Interestingly, lumichrome was correlated with the five year risk of onset in premanifest HD subjects (no correlation to ageing was found, r = 0.05, p-value = 0.76), but did not correlate with any other measure of disease severity. Lumichrome is part of the riboflavin metabolism where it is in strict equilibrium with the amount of riboflavin (vitamin B_2_), which is an essential compound in the brain that is transported from the blood through the CSF into the brain. Riboflavin has, as a majority of other findings herein, been connected to the energy metabolism where a riboflavin deficiency has resulted in a mitochondrial dysfunction^44^. Riboflavin is also essential for monoamine oxidase (MAO) activity^45^, which in its different isoforms is needed for metabolism of several important neurotransmitters such as norepinephrine, serotonin and dopamine. The changes in lumichrome levels might be directly connected to increased MAO-activity due to locally increased levels of dopamine^46^, but this finding may also indicate a deficiency in the energy metabolism before disease onset.

Taken together, these observations support the hypotheses of a negative energy balance in HD and mitochondrial dysfunction, previously supported by multiple studies^9,11,47–49^.

The most prominent limitation of the study was the limited number of mHTT carriers, which is a natural restraint of a single center HD study. This limitation suppresses the statistical power and confidence of our findings. Facing this limitation, the pathway analysis was advantageous as it couples statistical testing to molecular functioning, showing significance at a higher level^50^. To strengthen our findings further the metabolites showing altered levels were also associated to measures of disease severity, enabled by the well-characterized demographic data of the mHTT carriers. Moreover, to ensure reproducibility and extraction of variation true to the global HD population, profound cross-validation was performed to estimate more accurate AUROC values and VIP scores of the metabolites in the PLS-DA models. Finally, very few of the altered metabolites were found to change significantly over time. The limited number of mHTT carriers where a second sample was collected and the relative short time interval between samplings may be a reason to this.

Aside from the limited sample size, the study contained a number of other restraints. The natural age difference between premanifest and manifest HD subjects with a similar number of CAG repeats could be eliminated by age effect estimation and correction using the age matched controls. By such, we assumed that the mHTT carriers were depicting an accelerated effect caused by a combination of aging and neurodegeneration. To prevent removal of neurodegenerative alterations that are confounding with age and are of interest, we estimated the “normal” age effect in solely controls.

Moreover, the administration of drugs to some but not all subjects, could affect the results. Excluding patients on antidepressants and antipsychotics, univariate analyses of the altered metabolites between manifest and premanifest HD, as well as manifest HD compared with controls demonstrated similar changes but with less significant power, implying that the findings were not treatment specific (see Supplementary Table S5). However, as only the use of antidepressants and antipsychotics were considered, effects of other medications cannot be ruled out. Information regarding menopausal status of female patients as well as estrogen replacement therapy were neither collected. Trends of menopausal effects in the age of onset have been indicated^51^. However, stratification on presumed menopausal status (females <50 *versus* ≥50 years of age), resulted in only minority groups of presumed post-menopausal women (n_HD_ = 3, n_preHD_ = 1, n_control_ = 10). As such, Chi-squared tests comparing the distributions of menopausal status between diagnostic groups resulted in no significant differences (p-value > 0.05). But it cannot be ruled out that there may be metabolite expressions that are affected by menopausal status. Moreover, the inter-group *post hoc* comparisons could rule out gender, number of CAG repeats and sampling intervals as confounding factors.

Finally, one of the major challenges in metabolomics studies is metabolite identification. Identification by an *in house* library has the advantage of high reliability but the restraint of being limited to the composed compounds. The PCA model generated on the full metabolome suggested global biochemical differences between the three groups. But when limiting the data to metabolites with an identity, the segregations were not as clear, indicating that there are other biochemical alterations of importance that have not been fully revealed herein. The PLS-DA models extracted 24 and 28 metabolites each, comparing manifest HD with controls and manifest with premanifest HD, where 14 metabolites were in common. A majority (15 and 18 respectively) of the altered metabolites were found to be decreased in manifest compared with premanifest HD and controls, in agreement to what has previously been found in the proteome of HD CSF^13^.

In conclusion, we found tyrosine metabolism, including the dopaminergic species, to be altered in manifest HD patients. All corresponding metabolites from the tyrosine metabolism demonstrated moderate to strong associations to measures of disease severity and symptoms. Thyroxine showed particular promise as it was associated to all measures including the five year risk of onset in premanifest HD subjects. Moreover, a large proportion of the biochemical changes supported previous findings of an impaired energy metabolism in HD, where decreased levels of lumichrome were typical for all mHTT carriers. These findings are important steps towards characterizing the molecular pathogenesis in HD and support the hypothesis that the CSF metabolome may be used to explore changes caused by the HTT mutation, which may be essential for future development of novel disease-modifying therapies for HD.

## Supplementary information

Supplementary information

## Data Availability

The mass spectrometry data generated/analyzed in current study is available on the MetaboLights database, study identifier: MTBLS749.
