# Supplementary material for: Alterations in the tyrosine and phenylalanine pathways revealed by biochemical profiling in cerebrospinal fluid of Huntington’s disease subjects
Source: Sci Rep. 2019 Mar 11;9:4129. doi: 10.1038/s41598-019-40186-5 (PMC6411723; doi:10.1038/s41598-019-40186-5)
Supplement: Supplementary file 1 — Supplementary information [file 41598_2019_40186_MOESM1_ESM.pdf]

Supplementary information

Alterations in the tyrosine and phenylalanine pathways revealed by biochemical profiling in cerebrospinal fluid of Huntington's disease subjects

Stephanie Herman, Valter Niemelä, Payam Emami Khoonsari, Jimmy Sundblom, Joachim Burman, Anne-Marie Landtblom, Ola Spjuth, Dag Nyholm, Kim Kultima

Mass spectrometry analysis

The analysis was performed on a Thermo Ultimate 3000 HPLC and Thermo Q-Exactive Orbitrap mass spectrometer. 10 µL sample was injected to a Thermo Accucore aQ RP C18 column (100 × 2.1 mm, 2.6 µm particle size). The analytical gradient was initiated with an isocratic flow for 3 min (0% B), followed by 2.6 min (0-10% B), 8.3 min (10-100% B), 3 min (100% B) and finally re-equilibration of column for 3 min (0% B), where A is 0.1% formic acid in MilliQ water and B is 89.9% acetonitrile, 10% isopropanol and 0.1% formic acid. Mass spectrometry data were acquired in profile mode (in positive and negative ion mode), using a mass range of 70-900 m/z in the first 5 min and 148-900 m/z in the following 15 min (to avoid low mass contaminants) in the positive ion mode and 70-900 m/z throughout in negative ion mode. The spray voltage on the heated electrospray source was set to 4.0kv and capillary temperature of 350 °C. The resolution used was set to 70k, the automatic gain control (AGC) target and maximum ion injection time were set to 1e6 and 250 ms, respectively. MS/MS spectra were collected in positive and negative ionization mode where the five most abundant ions were collected using an AGC of 2e5, an isolation width of 1.2 mass over charge (m/z) and fragmented using a normalized collision energy of 30, measured at a resolution of 35k and maximum IT of 250 ms. The exclusion time was set to 20 s.

Table S1. Non-default parameter values used for pre-processing in KNIME. For all parameters not mentioned, the default values were used.

Parameter	Value
FeatureFinderMetabo	
chrom_peak_snr	2.0
min_trace_length	1.0
enable_RT_filtering	false
isotope_filtering_model	none
report_convex_hulls	true
FeatureLinkerUnlabeledQT	
nr_partitions	10
ignore_charge	true
max_difference (distance_RT)	10.0
max_difference (distance_MZ)	5.0
unit	ppm

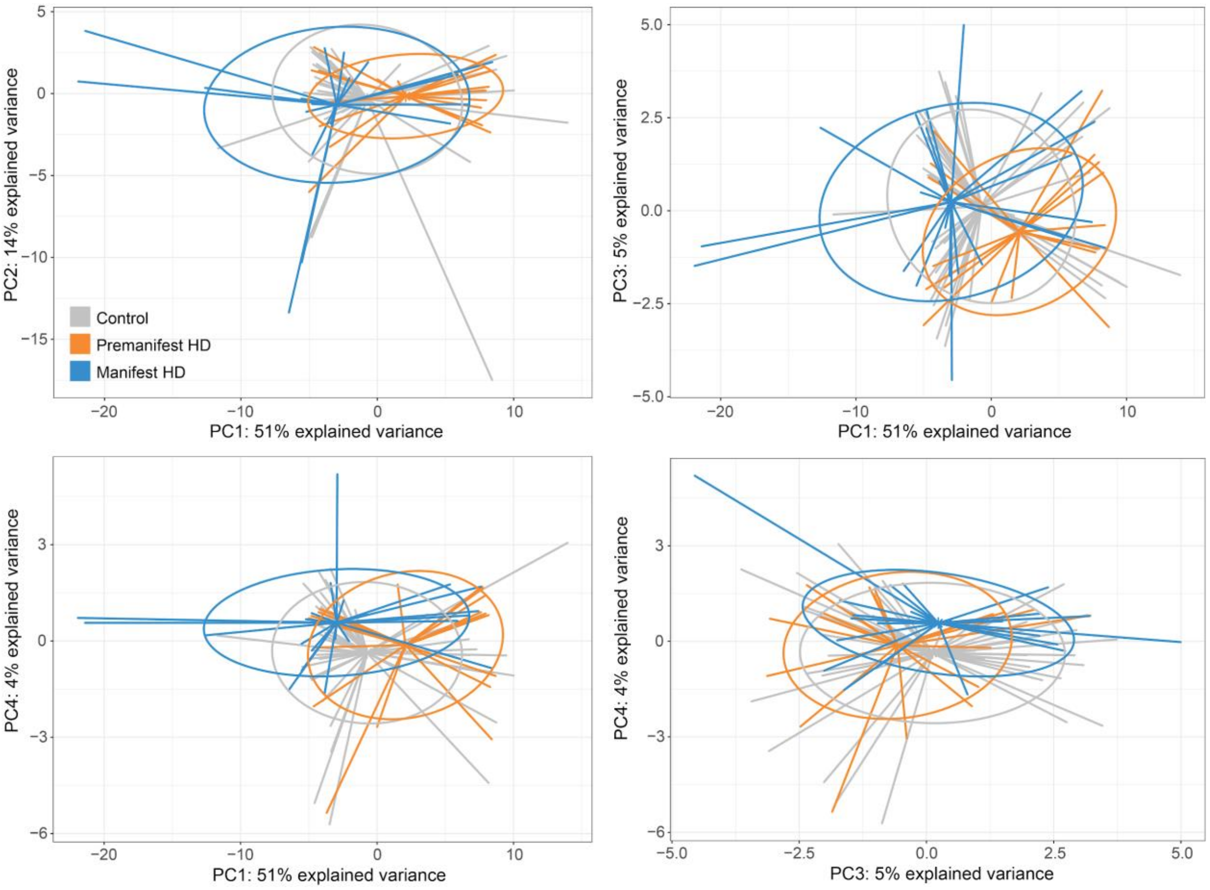

Figure S1. Age-adjusted principal component analysis on the 94 identified metabolites.

Table S2. Results from the pathway analysis based on the altered metabolites in manifest HD patients compared with controls.

Pathway	Coverage	p-value	FDR	Impact
Aminoacyl-tRNA biosynthesis	5/75	**$2.1×10^{-4}$**	**0.013**	0.0
Phenylalanine metabolism	4/45	**$3.4×10^{-4}$**	**0.013**	0.173
Valine, leucine and isoleucine biosynthesis	3/27	**$1.1×10^{-3}$**	**0.029**	0.040
Valine, leucine and isoleucine degradation	3/40	**$3.4×10^{-3}$**	**0.069**	0.022
Purine metabolism	4/92	**$5.0×10^{-3}$**	**0.080**	0.048
Phenylalanine, tyrosine and tryptophan metabolism	2/27	**0.018**	0.246	0.008
Nitrogen metabolism	2/39	**0.037**	0.420	0.0
Pyrimidine metabolism	2/60	0.080	0.797	0.024
Tyrosine metabolism	2/76	0.120	0.974	0.110
Arginine and proline metabolism	2/77	0.122	0.974	0.006
Caffeine metabolism	1/21	0.154	1.0	0.031
Thiamine metabolism	1/24	0.174	1.0	0.0
Steroid hormone biosynthesis	2/99	0.182	1.0	0.037
Pantothenate and CoA biosynthesis	1/27	0.194	1.0	0.0
Propanoate metabolism	1/35	0.244	1.0	0.0
Ubiquinone and other terpenoid-quinone biosynthesis	1/36	0.250	1.0	0.0

Table S3. Results from the pathway analysis based on the altered metabolites in manifest HD patients compared with premanifest HD subjects.

Pathway	Coverage	p-value	FDR	Impact
Aminoacyl-tRNA biosynthesis	6/75	**2.8×10^{-5}**	**2.2×10^{-3}**	0.0
Phenylalanine, tyrosine and tryptophan biosynthesis	3/27	**1.5×10^{-3}**	**0.039**	0.008
Valine, leucine and isoleucine biosynthesis	3/27	**1.5×10^{-3}**	**0.039**	0.040
Tyrosine metabolism	4/76	**3.7×10^{-3}**	**0.061**	0.182
Nitrogen metabolism	3/39	**4.3×10^{-3}**	**0.061**	0.0
Valine, leucine and isoleucine degradation	3/40	**4.6×10^{-3}**	**0.061**	0.022
Phenylalanine metabolism	3/45	**6.4×10^{-3}**	**0.073**	0.173
Purine metabolism	4/92	**7.3×10^{-3}**	**0.073**	0.059
Arginine and proline metabolism	2/77	0.144	1.0	0.010
Caffeine metabolism	1/21	0.169	1.0	0.031
Thiamine metabolism	1/24	0.190	1.0	0.0
Pantothenate and CoA biosynthesis	1/27	0.212	1.0	0.0
Propanoate metabolism	1/35	0.266	1.0	0.0
Ubiquinone and other terpenoid-quinone biosynthesis	1/36	0.272	1.0	0.0
Lysine degradation	1/47	0.340	1.0	0.018
Glycine, serine and threonine metabolism	1/48	0.346	1.0	0.0
Cysteine and methionine metabolism	1/56	0.391	1.0	0.101
Tryptophan metabolism	1/79	0.505	1.0	0.109
Steroid hormone biosynthesis	1/99	0.588	1.0	0.011

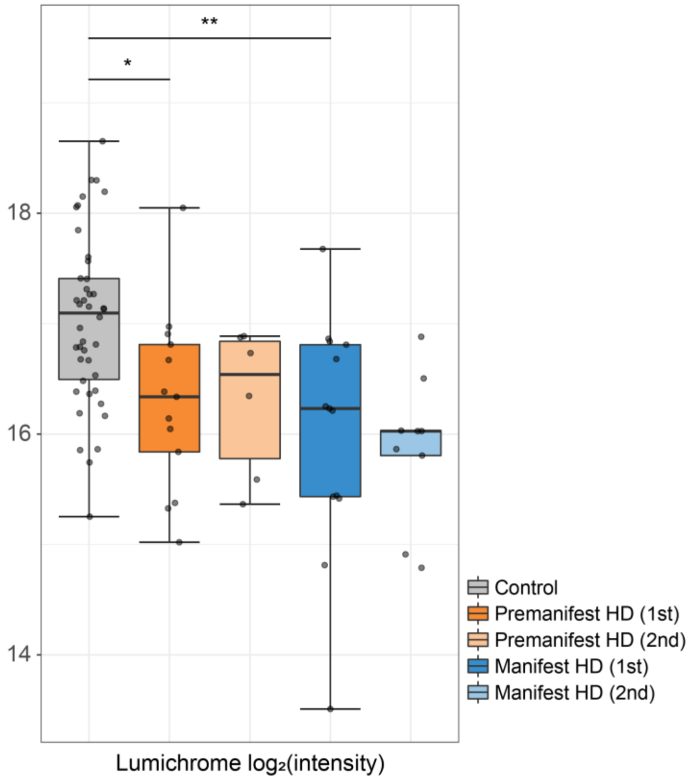

Figure S2. Cerebrospinal fluid levels of lumichrome. *, p<0.05; **, p<0.01; and ***, p<0.001.

Table S4. Correlation between the CSF levels of metabolites and measures of disease severity, disease burden score (DBS) and the five year risk of onset. Statistically significant correlations (p-value<0.05) have been bolded. DBS: disease burden score; TFC: total functional capacity; 5yrisk: 5 year risk of onset; TMS: total motor score; SC: Stroop color; SWR: Stroop word reading; SI: Stroop interference; CVF: category verbal fluency; VFL: verbal fluency letters; SDMT: symbol digit modalities test. *Metabolite with a demonstrated age dependence that have been age corrected.

Metabolite	DBS	TFC	5yrisk	TMS	SC	SWR	SI	CVF	VFL	SDMT
L-DOPA	-0.30	**0.43**	0.28	**-0.47**	**0.50**	**0.47**	**0.59**	**0.56**	**0.43**	**0.48**
Xanthine*	**0.40**	**-0.55**	-0.10	**0.48**	**-0.43**	**-0.40**	-0.38	**-0.51**	**-0.46**	**-0.45**
Ophthalmate	**-0.50**	**0.48**	-0.20	**-0.54**	**0.57**	**0.44**	**0.60**	**0.53**	**0.41**	**0.45**
Creatinine	**0.40**	**-0.49**	0.15	**0.55**	**-0.46**	**-0.43**	**-0.47**	**-0.48**	**-0.48**	**-0.52**
Tyrosine*	-0.25	0.25	-0.38	**-0.42**	0.37	0.29	**0.49**	**0.43**	0.26	**0.41**
5-hydroxytryptophan	**-0.47**	**0.60**	-0.35	**-0.46**	**0.56**	**0.57**	**0.50**	**0.42**	**0.48**	**0.53**
Adenosine	0.38	-0.22	0.01	0.34	-0.39	-0.29	**-0.43**	-0.33	-0.21	**-0.45**
Phenylalanine	-0.30	0.11	0	-0.33	0.33	0.23	**0.41**	**0.54**	0.30	0.38
Phenylacetate	0.38	-0.37	-0.02	**0.40**	-0.33	-0.31	-0.27	-0.29	-0.37	**-0.47**
Thyroxine	**-0.43**	**0.54**	**-0.73**	**-0.48**	**0.58**	**0.50**	**0.43**	**0.49**	**0.42**	**0.61**
Glutarylcarnitine*	-0.29	**0.44**	-0.20	-0.36	0.38	0.34	0.36	**0.50**	0.33	0.37
O-succinyl-homoserine*	**-0.45**	0.34	-0.49	-0.33	**0.40**	0.30	0.38	0.38	0.26	0.26
Isoleucine	0.06	**0.58**	0.51	-0.35	0.34	0.32	0.30	0.28	0.34	0.40
Aldosterone/cortisone	-0.18	0.36	0.05	-0.36	0.33	0.34	**0.44**	0.41	0.26	0.27
O-acetylcarnitine	-0.26	0.27	0.17	-0.29	0.28	0.30	0.25	0.20	**0.42**	0.32
4-acetamidobutanoate*	**0.41**	-0.32	-0.07	**0.39**	-0.23	-0.26	-0.21	-0.25	-0.31	-0.22
N,N,N-trimethyllysine	0.31	-0.19	-0.06	0.27	-0.38	-0.36	**-0.42**	-0.25	-0.17	-0.29
Dopamine	-0.37	0.36	**-0.62**	**-0.48**	**0.51**	**0.49**	**0.44**	0.34	0.39	**0.44**
Lumichrome	-0.14	0.06	**-0.59**	-0.04	0.18	0.23	0.12	0.07	-0.03	0.13

Table S5. Table of altered metabolites with a VIP>1.0 from the full PLS-DA models comparing manifest HD with premanifest and controls respectively. Univariate analyses have been done, excluding subjects on antidepressants or antipsychotics, extracting log_2 fold change (FC) values and significance levels (p-values and FDR values corrected for multiple comparisons). Contradictionary to previous log_2 FC values have been marked red and comparisons with a p-value<0.1 have been bolded.

Metabolite	FC HD-C	p-value HD-C	FDR HD-C	FC HD-pHD	p-value HD-pHD	FDR HD-pHD
Tyrosine*	-0.09	0.504	0.728	-0.19	0.181	0.413
Creatinine	-0.02	0.850	0.884	0.10	0.429	0.690
Phenylalanine	-0.05	0.489	0.728	**-0.14**	**0.095**	**0.413**
Leucine	-0.11	0.222	0.601	-0.08	0.358	0.640
Lumichrome	-1.24	0.111	0.413	-	-	-
5-methylcytosine*	**0.32**	**0.031**	**0.413**	-	-	-
Glutarylcarnitine*	0.08	0.606	0.751	-0.01	0.945	0.945
Ophthalmate	-0.22	0.575	0.747	-0.65	0.156	0.413
Xanthine*	0.13	0.231	0.601	0.15	0.185	0.413
Corticosterone	-0.09	0.885	0.885	-	-	-
L-DOPA	-0.23	0.289	0.653	**-0.45**	**0.073**	**0.413**
Salicylate	-0.28	0.443	0.720	-	-	-
Phenylacetate	0.06	0.671	0.793	0.11	0.503	0.729
N-acetylproline*	0.25	0.108	0.413	-	-	-
Phosphocreatine	0.22	0.228	0.601	-	-	-
1-methyladenosine	-0.11	0.843	0.884	-	-	-
Hypoxanthine	**0.24**	**0.085**	**0.413**	0.21	0.140	0.413
Aldosterone/cortisone	**-0.32**	**0.055**	**0.413**	-0.20	0.113	0.413
Deoxyuridine	-0.28	0.302	0.653	-	-	-
Valine	**-0.20**	**0.037**	**0.413**	**-0.16**	**0.073**	**0.413**
Isoleucine	-0.10	0.555	0.747	0.03	0.834	0.864
Inosine	**0.27**	**0.079**	**0.413**	-	-	-

Cyclic AMP	0.24	0.353	0.656	-	-	-
O-succinyl-homoserine*	-0.35	0.331	0.656	-0.36	0.344	0.640
Tryptophan[+]	-	-	-	-0.19	0.119	0.413
O-acetylcarnitine	-	-	-	-0.11	0.640	0.742
N,N,N-trimethyllysine	-	-	-	**0.32**	**0.069**	**0.413**
Adenine	-	-	-	**0.29**	**0.036**	**0.413**
S-adenosylhomocysteine*	-	-	-	0.15	0.536	0.729
4-acetamidobutanoate*	-	-	-	0.06	0.694	0.774
Thyroxine	-	-	-	-0.10	0.553	0.729
4-quinolinecarboxylate	-	-	-	-0.19	0.375	0.640
Adenosine	-	-	-	0.39	0.223	0.462
Dopamine	-	-	-	-0.12	0.472	0.720
5-hydroxytryptophan	-	-	-	-0.21	0.603	0.729
N-acetylalanine	-	-	-	0.03	0.727	0.781
Tryptophan[-]	-	-	-	-0.17	0.171	0.413
DHEAS	-	-	-	-0.42	0.581	0.729
